# Interleukin-17A Mediates Hippocampal Damage and Aberrant Neurogenesis Contributing to Epilepsy-Associated Anxiety

**DOI:** 10.3389/fnmol.2022.917598

**Published:** 2022-07-06

**Authors:** In-Young Choi, Mi-La Cho, Kyung-Ok Cho

**Affiliations:** ^1^Department of Pharmacology, College of Medicine, The Catholic University of Korea, Seoul, South Korea; ^2^Department of Medical Life Science, College of Medicine, The Catholic University of Korea, Seoul, South Korea; ^3^Department of Biomedicine and Health Sciences, The Catholic University of Korea, Seoul, South Korea; ^4^Catholic Neuroscience Institute, The Catholic University of Korea, Seoul, South Korea; ^5^Institute for Aging and Metabolic Diseases, The Catholic University of Korea, Seoul, South Korea

**Keywords:** IL-17A, epilepsy, aberrant neurogenesis, anxiety, neuronal death, hippocampus, seizures

## Abstract

Anxiety disorder is one of the most common comorbidities in temporal lobe epilepsy (TLE), but its neurobiological mechanisms remain unclear. Here we identified a novel target, interleukin-17A (IL-17A), which can contribute to TLE-associated anxiety. Epileptic seizures were induced in 6-week-old IL-17A wild-type (WT) and knockout (KO) mice by pilocarpine injection. To evaluate anxiety level, we subjected mice to open field and elevated plus maze (EPM) tests and measured the time animals spent in center zone or open arms. Epileptic IL-17A WT mice showed thigmotaxis and reluctance to stay in open arms, whereas IL-17A KO mice spent more time in the center area and open arms, suggesting alleviated anxiety in epilepsy. Histological assessments revealed that hippocampal neuronal death as evaluated by Fluoro-Jade B staining was significantly reduced in IL-17A KO mice. Moreover, at 6 weeks after pilocarpine-induced status epilepticus, the number of hilar ectopic granule cells was also markedly decreased by IL-17A deficiency without a difference in the proliferation of neural progenitors or the generation of newborn neurons in the dentate gyrus. Taken together, our data demonstrated that IL-17A deletion mitigates TLE-associated anxiety behavior, possibly via the hippocampal neuroprotection and the reduction of seizure-induced aberrant neurogenesis.

## Introduction

Temporal lobe epilepsy (TLE) is one of the most prevalent acquired epilepsies in adults (Tellez-Zenteno and Hernandez-Ronquillo, 2012). Patients with TLE often exhibit various psychiatric comorbidities including depression, anxiety, and psychotic disorders (Lin et al., 2012; Vinti et al., 2021). Among the comorbidities, anxiety disorder is the most common epilepsy-associated comorbid conditions (Brandt et al., 2010; Jansen et al., 2019) and leads to a severe reduction in quality of life and poor prognosis in terms of responsiveness to pharmacotherapy (Choi-Kwon et al., 2003; Johnson et al., 2004; Petrovski et al., 2010). Despite the high incidence of comorbid anxiety, its neurobiological mechanisms have not been fully elucidated.

The hippocampus has a central role in managing anxiety and stress (Engin et al., 2016; Jimenez et al., 2018). Adult-generated neurons are also involved in the control of anxiety; suppressing hippocampal neurogenesis increases anxiety-like behaviors (Revest et al., 2009) whereas enhancing neurogenesis promoted resilience to stress-related increased anxiety (Hill et al., 2015). However, the contribution of hippocampal lesions to the development of anxiety disorder is controversial (Bannerman et al., 2002; Kjelstrup et al., 2002; Cominski et al., 2014). Additionally, despite the prominence of injury-induced abnormal hippocampal neurogenesis in many central nervous system (CNS) diseases including epilepsy (Parent et al., 2006; Cho and Kim, 2010; Niv et al., 2012; Cho et al., 2015; Ibrahim et al., 2016), studies demonstrating the impact of aberrant neurogenesis on epilepsy-associated anxiety are scarce.

Interleukin-17A (IL-17A) is a cytokine that belongs to the IL-17 family of cytokines, which consists of six members: IL-17A, IL-17B, IL-17C, IL-17D, IL-17E, and IL-17F (McGeachy et al., 2019). Multiple cell types such as Th17, γδ T cells, neutrophils, astrocytes, and microglia produce IL-17A and this cytokine serves versatile functions in adult hippocampal neurogenesis and anxiety-like behaviors (Liu et al., 2014; Lin et al., 2016; Tfilin and Turgeman, 2019; Alves de Lima et al., 2020; Milovanovic et al., 2020). Interestingly, IL-17A level is increased in the serum, cerebrospinal fluid, and brain of TLE patients (Mao et al., 2013; Wang et al., 2015; He et al., 2016). The level of IL-17A is also reported to be higher in patients with generalized anxiety disorder (Vieira et al., 2010), suggesting a potential role of IL-17 in epilepsy and anxiety disorder. Taken together, these findings raise the possibility that IL-17A in epilepsy may contribute to anxiety disorder via the regulation of hippocampal neurogenesis. Thus, in the present study, we investigated the role of IL-17A in epilepsy-associated anxiety and its biological mechanisms, using IL-17A wildtype (WT) and IL-17A knockout (KO) mice subjected to pilocarpine-induced status epilepticus (SE).

## Materials and Methods

### Mice

IL-17A KO mice (mixed background crossed to C57BL/6 for more than 5 generations) were a gift from Ishigame et al. (2009). IL-17A^±^ mice were crossed to obtain IL-17^–/–^ (WT) and IL-17^–/–^ (KO) mice. Mice were genotyped by polymerase chain reaction (PCR) using primers for WT (ACT CTT CAT CCA CCT CAC ACG A, CAG CAT CAG AGA CTA GAA GGG A) and KO (GCC ATG ATA TAG ACG TTG TGG C, CAG CAT CAG AGA CTA GAA GGG A) mice. All experimental procedures were approved by the Institutional Animal Use and Care Committee at the Catholic University of Korea (approval number: CUMS-2020-0342-01, 2020-0103-04) and were performed in accordance with guidelines by the National Institutes of Health. All mice were bred and housed in an animal facility with a 12 h light, 12 h dark cycle and access to food and water *ad libitum*.

### Pilocarpine-Induced Mouse Model of Epilepsy

Epileptic seizures were induced by pilocarpine injection as described in our previous work (Jeong et al., 2011; Cho et al., 2019; Choi et al., 2021). In brief, to minimize the peripheral cholinergic side effects of pilocarpine, 6-week-old IL-17A WT and KO mice were first injected with scopolamine methyl nitrate (i.p.; 2 mg/kg; Sigma-Aldrich S2250) and terbutaline hemisulfate salt (i.p.; 2 mg/kg; Sigma-Aldrich T2528) in 0.9% saline. After 30 min, pilocarpine (Sigma-Aldrich P6503) prepared in normal saline was injected intraperitoneally (i.p., 240 mg/kg for male, 260 mg/kg for female) to induce SE. Since females are supposed to have higher resistance to pilocarpine-induced SE (Scharfman et al., 2005; Scharfman and MacLusky, 2014), we used different doses for male and female. Mice were then placed in an incubator maintained at 31°C (ThermoCare). The seizure stage was evaluated using a modified Racine scale (stage 1, facial clonus; stage 2, head nodding; stage 3, forelimb clonus; stage 4, rearing; stage 5, rearing and falling). Animals consistently showing stage 3–5 were defined as SE and included in the study. We confirmed that acute seizure severity such as the onset of the first seizure (WT: male 21.540 ± 2.303 vs. female: 20.000 ± 3.873; KO: male 14.900 ± 1.779 vs. female 21.000 ± 4.276) and SE (WT: male 32.290 ± 1.936 vs. female 32.800 ± 4.140; KO: male 26.700 ± 2.422 vs. female 30.400 ± 6.742) was comparable between male and female per each genotype (Supplementary Figures 1A,B). At 3 h after SE, diazepam was injected (i.p.; 10 mg/kg; Sigma-Aldrich D0899) to calm behavioral seizures, followed by administration of a single dose of 5% dextrose (i.p.; 1 ml) to promote recovery. All experimental animals were placed in an incubator (28–30°C) and given water-soaked foods for 2 days until they gained weight. For the sham group, animals were injected with the same doses of scopolamine and terbutaline as above but treated with saline instead of pilocarpine.

### Behavioral Tests

#### Open Field Test

Six weeks after pilocarpine injection, mice were placed in a square white box (44 cm × 44 cm × 30 cm) and allowed to roam freely for 15 min while recording was carried out by a video camera on the ceiling. All trials were performed under dim light (60 lux) between 7 am and 9 am by the same researcher. For habituation, mice were single housed for 1 week at 5 weeks after SE, and they were transferred to the behavior room 1 day before the experiment. To assess anxiety-like behaviors, the percentage of time spent in the central zone (20 cm × 20 cm) and the peripheral zone divided by the total time was calculated using SMART software (Panlab Harvard Apparatus) (Ryu et al., 2018). The animal’s location in the open field arena was determined by tracking the center of mass of each mouse. Total distance moved during the 15-min trial was also determined for each mouse to evaluate locomotor activity.

#### Elevated Plus Maze Test

One day after OFT, mice were placed in the center of an elevated plus maze (EPM) with two open and two closed arms with each arm of the maze 5 cm wide, 35 cm long, and 75 cm high. The closed arms were covered by perimeter walls 20 cm high. For 5 min, animals were allowed to freely roam in the EPM under the light intensity at 60 lux and returned to their home cages after the experiments. To assess anxiety level, the percentage of time spent in the open arms out of the total time was calculated using the same tracking tool of SMART software.

### Video/Electroencephalogram Monitoring

Video/Electroencephalogram (EEG) recording was conducted according to previously published studies (Brulet et al., 2017; Kim and Cho, 2018). At 5 weeks after SE, epidural electrodes were stereotaxically implanted at anteroposterior (AP) + 0.1 mm, mediolateral (ML) + 0.1 mm (reference), and AP –0.2 mm, ML + 0.22 mm (cortical electrode) from bregma. The recording electrodes were connected to a wireless EEG transmitter (TA11ETAF10; Data Sciences International, Inc.) placed subcutaneously on the animals’ backs. After another week of recovery from the electrode implantation surgery, spontaneous recurrent seizures (SRS) were monitored 24 h/day for up to 14 days by analyzing the frequency of SRS and duration of each seizure. Convulsive seizures were defined by repetitive epileptiform spiking (≥ 3 Hz) that persisted for more than 3 s with confirmation by video recordings.

### Histologic Assessments

As the proliferative activity of hippocampal progenitors and aberrant neurogenesis have been escalated at 3 days and 14–56 days after SE (Parent et al., 1997; Parent et al., 2006; Walter et al., 2007), respectively, we decided to collect the brain samples at 3 or 42 d after pilocarpine-induced SE. Mice were anesthetized by administering a mixture of ketamine (50 mg/ml) and xylazine (23.3 mg/ml) solution (4:0.5) at a dose of 10 ml/kg body weight. Mice were perfused transcardially with normal saline. The brain was removed and fixed with 4% paraformaldehyde (PFA) in 0.1 M phosphate-buffered saline (PBS) for 3 days, followed by the cryoprotection in 30% sucrose in 0.1 M PB. The brains were bisected and the half-brains were cut coronally (30 μm thick) using a cryostat.

Fluoro-Jade B (FJB) staining for evaluating degenerating neurons was performed using samples at 3 d after SE. Hippocampal brain sections were mounted on microscope slides. Slides were placed in distilled water for 1 min and samples were oxidized by soaking in a solution of 0.06% KMnO_4_ for 7 min. Sections were stained with 0.001% FJB (Biosensis, TR-150-FJB) in 0.1% acetic acid for 30 min, rinsed with distilled water 3 times for 1 min, and dried for 1 h. Slides were dehydrated with xylene and covered with dibutylphthalate polystyrene xylene (DPX) mounting medium. Images were obtained with Lionheart FX (Biotek).

For immunohistochemical staining, free-floating methods were applied as previously described (Jang et al., 2019; Cho et al., 2020). Briefly, the sections washed in 0.01 M PBS, and then placed in 3.5% hydrogen peroxide solution at room temperature for 15 min to remove endogenous peroxidase activity. After washing with 0.01 M PBS, the sections were incubated in a blocking solution containing 3% normal goat serum and 0.3% Triton X-100 for 1 h, followed by incubation with the following primary antibodies at 4°C overnight: rabbit anti-Ki67 (1:500, Santa Cruz Biotechnology, sc-23900), guinea pig anti-doublecortin (DCX, 1:1,000, Millipore, AB2253), rabbit anti-Prox1 (1:2,000, Millipore, ABN278), rabbit anti-ionized calcium binding adaptor molecule 1 (Iba1, 1:500, Wako, 019-19741), and mouse anti-glial fibrillary acidic protein (GFAP, 1:1,000, Millipore, MAB360). The next day, after washing with 0.01 M PBS, the sections were incubated with peroxidase-conjugated secondary antibodies for 2 h at room temperature. The sections were incubated with 0.05% 3,3′-diaminobenzidine (DAB) solution and mounted with DPX. Imaging was carried out under an optical microscope (BX51; Olympus).

### Microscope Analysis and Quantification

Immunoreactive or FJB-positive cells were quantified as previously described (Brulet et al., 2017). The subgranular zone (SGZ) was defined as the area within the average diameter of one granule cell from the inner edge of the granule cell layer (GCL). The hilus was defined as a triangular area connecting the end points of the upper and lower blades of GCL, excluding SGZ. The CA1 and CA3 pyramidal cell layer (PCL) were determined by the PCL thickness and the soma size, as CA1 PCL is thinner and CA3 neurons have relatively larger soma. The number of each marker-positive cell was counted in every 12th coronal section throughout the hippocampus; the values were added and multiplied by 24 to estimate the total number of cells in each animal. For reactive gliosis,% Iba1- or GFAP-immunoreactive areas in the dentate gyrus (DG), CA1 and CA3 subfields of the hippocampus were quantitatively analyzed using NIH ImageJ software. Briefly, the pixel intensity of the non-tissue area in each image was measured as the background intensity. Using the threshold function, the area of pixels greater than the background intensity was assessed as Iba1- or GFAP-immunoreactive area, which was divided by the measurement of the pixel area of DG, CA1, and CA3 subfields of the hippocampus.

### Statistical Analysis

Data are presented as mean ± standard error of the mean (SEM) and statistical significance was assessed using GraphPad Prism 9 software (GraphPad Software Inc.). Experimental groups were randomly assigned, and the exact sample size is presented in the figure legends. To reduce the experimental bias, behavioral tests were performed by a researcher blind to the genotype. Data obtained from both male and female were analyzed together unless specifically indicated otherwise. Statistical differences were determined by two-tailed Student’s unpaired *t*-test for data with equal variances and two-tailed Student’s unpaired *t*-test with Welch’s correction if the variance was significantly different. If a normal distribution was not assumed, Mann–Whitney *U*-test was performed. *p* < 0.05 was considered significant.

## Results

### Interleukin-17A Deletion Ameliorates Epilepsy-Associated Anxiety

**FIGURE 1:**
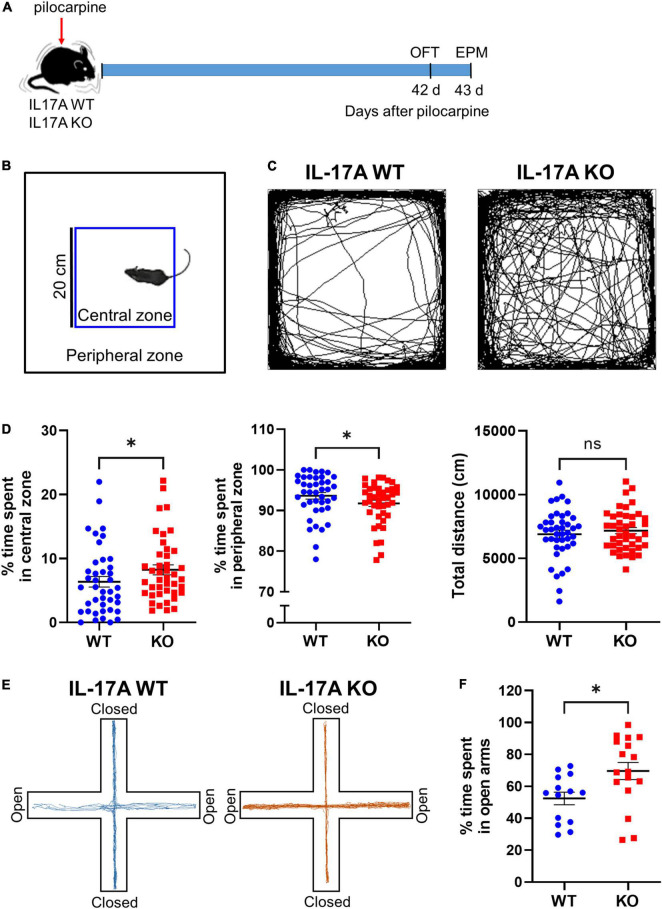
IL-17A deficiency attenuates anxiety-like behaviors in chronic epilepsy in mice. **(A)** Timeline showing the experimental design. **(B)** Schematic presentation of central and peripheral zones in the open field arena. **(C)** Representative traces of IL-17A WT and KO mice showing spontaneous movement in an open field box. **(D)** Graphs showing the percentage of time spent in the central zone and the peripheral zone and the total distance traveled in the open field. *n* = 41 (WT) and *n* = 42 (KO). Detailed statistics are as follows. Central zone: Mann–Whitney *U*-test, *p* = 0.044, *U* = 640.500; peripheral zone: Mann–Whitney *U*-test, *p* = 0.044, *U* = 640,500; total distance: Student’s unpaired *t-*test, *p* = 0.481, *t*(81) = 0.707. **(E)** Representative traces of IL-17A WT and KO mice showing spontaneous movement in an elevated plus maze. **(F)** A graph showing the percentage time spent in the open arms of the elevated plus maze. *n* = 14 (WT) and *n* = 17 (KO). Student’s unpaired *t-*test, *p* = 0.019, *t*(29) = 2.492. Data are presented as mean ± SEM. *p < 0.05, NS, not significant.

To investigate the role of IL-17A in chronic epilepsy-related anxiety, IL-17A WT and KO mice were subjected to an open field test (OFT) and EPM test at 6 weeks after pilocarpine-induced SE (Figure 1A). After defining the central and peripheral zone of the open field arena (Figure 1B), we assessed the percentage of time that animals spent in the central and peripheral zones (Figures 1C,D). Epileptic IL-17A KO mice spent more time in the center (WT: 6.363 ± 0.826 vs. KO: 8.229 ± 0.779) and avoided the periphery (WT: 93.640 ± 0.827 vs. KO: 91.770 ± 0.779) of the open field arena compared with IL-17A WT mice (Figures 1C,D). However, the total distance moved in the open field (WT: 6,896 ± 305.100 vs. KO: 7,172 ± 244.400) was comparable between IL-17A WT and KO mice (Figure 1D), indicating no difference in locomotor activity.

To confirm the anti-anxiety effect of IL-17A deficiency in chronic epilepsy, we employed another widely used behavioral assay for testing anxiety status, the EPM test. In agreement with the OFT, epileptic IL-17A KO mice exhibited significantly more exploration time in the open arms compared with the WT group (WT: 52.420 ± 3.959 vs. KO: 69.630 ± 5.345; Figures 1E,F). However, when we evaluated the anxiety-like behaviors between male and female mice per genotype, there was no significant gender differences in both OFT (WT for central zone: male 6.018 ± 1.225 vs. female: 7.708 ± 1.279; WT for peripheral zone: male 93.980 ± 1.226 vs. female 92.290 ± 1.279; KO for central zone: male 6.024 ± 1.315 vs. female: 9.516 ± 1.338; KO for peripheral zone: Male 93.980 ± 1.314 vs. female 90.480 ± 1.337) and EPM (WT: male 55.000 ± 4.911 vs. female: 50.980 ± 5.674; KO: male 77.630 ± 7.813 vs. female 65.200 ± 7.458; Supplementary Figures 1C–F). Moreover, when we assessed the anxiety-like behaviors in sham IL-17A WT and KO mice, there was no significant difference in the time spent in the central zone (WT: 10.450 ± 0.888 vs. KO: 12.910 ± 1.000), peripheral zone (WT: 89.550 ± 0.888 vs. KO: 87.090 ± 1.000) of the open field arena and the open arms in EPM (WT: 33.820 ± 4.850 vs. KO: 40.910 ± 5.993; Supplementary Figure 2), demonstrating that the basal anxiety level was not altered by IL-17A KO. Taken together, these results suggest that deletion of IL-17A could attenuate anxiety-like behaviors in chronic epilepsy.

### Interleukin-17A Deletion Does Not Alter the Formation of Spontaneous Recurrent Seizures

**FIGURE 2:**
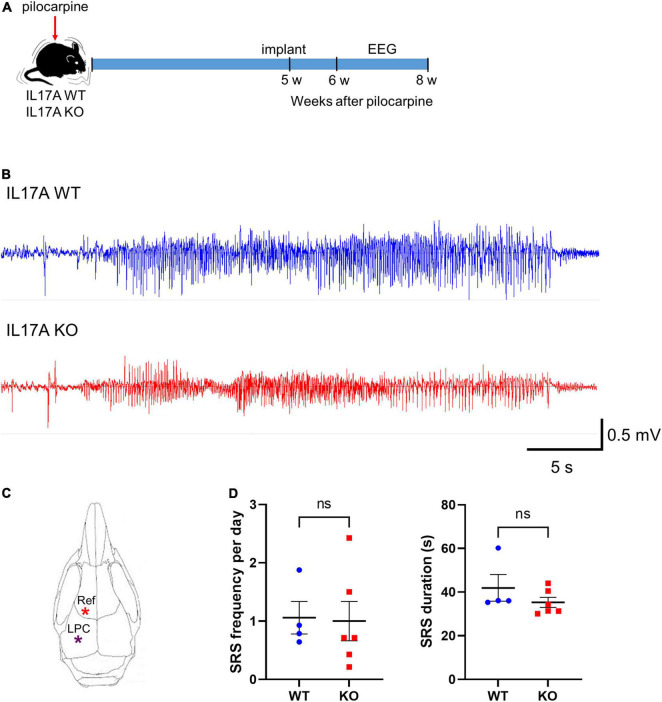
IL-17A deficiency in mice does not alter epileptogenesis. **(A)** Timeline showing the experimental design. **(B)** Representative EEG traces showing generalized seizures from IL-17A WT and IL-17A KO animals. **(C)** Schematic drawing of the mouse brain with locations of electrodes. Ref: reference; LPC: left parietal cortex. **(D)** Graphs showing the number of spontaneous recurrent seizures (SRS) per day and duration of each seizure. There was no difference in SRS frequency or duration between IL-17A WT and KO mice. *n* = 4 (WT) and *n* = 6 (KO). Detailed statistics are as follows. SRS frequency: Student’s unpaired *t-*test, *p* = 0.906, *t*(8) = 0.122; SRS duration: Mann–Whitney *U*-test, *p* = 0.257, *U* = 6,000. Data are presented as mean ± SEM. *p < 0.05, NS, not significant.

We next assessed the effect of IL-17A deletion on epileptogenesis by performing continuous video-EEG monitoring of epileptic IL-17A WT and KO mice. Five weeks after pilocarpine-induced SE, mice were implanted with two cortical epidural electrodes, one placed over the hippocampus (left parietal cortex; LPC) and the other over the olfactory bulb (reference; Ref) for wireless EEG recording (Figures 2A,C). One week later, freely moving mice were subjected to video-EEG recording for 2 weeks (Figure 2A). IL-17A deletion did not profoundly affect SRS frequency (WT: 1.058 ± 0.279 vs. KO: 1.000 ± 0.337) or the total duration of each seizure event (WT: 41.900 ± 6.101 vs. KO: 35.270 ± 2.322; Figures 2B,D). These results indicate no significant impact on epileptogenesis by IL-17A deletion.

### Interleukin-17A Deletion Protects Pilocarpine-Induced Hippocampal Neuronal Death Without Altering Reactive Gliosis in General

**FIGURE 3:**
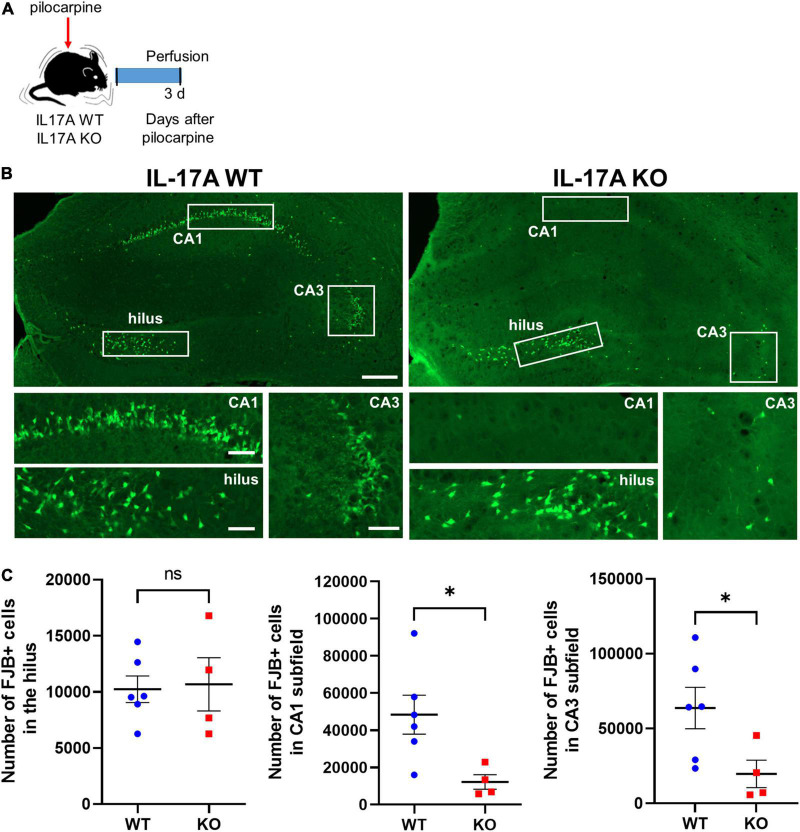
IL-17A deficiency in mice promotes neuroprotection against pilocarpine-induced status epilepticus (SE). **(A)** Timeline showing the experimental design. **(B)** Representative microscope images showing degenerating neurons stained with Fluoro-Jade B (FJB). Magnified image areas in the hilus, CA1, and CA3 subfields are indicated as white rectangles. Scale bar = 200 μm for low magnified images, 50 μm for higher magnified images. **(C)** Graphs showing the number of FJB-positive cells in the hilus, CA1, and CA3 subfields of the hippocampus. Fewer degenerating neurons were observed in CA1 and CA3 subfields of the hippocampus in IL-17A KO mice compared with the WT group. *n* = 6 (WT) and *n* = 4 (KO). Detailed statistics are as follows. Hilus: Mann–Whitney *U*-test, *p* = 0.971, *U* = 11,500; CA1: Mann–Whitney *U*-test, *p* = 0.019, *U* = 1,000; CA3: Mann–Whitney *U*-test, *p* = 0.038, *U* = 2,000. Data are presented as mean ± SEM. *p < 0.05, NS, not significant.

We then investigated histologic alterations induced by IL-17A deficiency after SE. FJB staining, which specifically labels degenerating neurons, clearly showed dying hippocampal cells at 3 d after pilocarpine-induced SE (Figures 3A,B). The number of FJB-positive neurons in CA1 (WT: 48,364 ± 10,488 vs. KO: 12,186 ± 3,921) and CA3 (WT: 63,676 ± 13,827 vs. KO: 19,650 ± 9,176) subfields of the hippocampus was markedly reduced in IL-17A KO mice compared with the IL-17A WT group, without a difference in the hilar neuronal death (WT: 10,232 ± 1,181 vs. KO: 10,668 ± 2,368; Figure 3C). Interestingly, FJB-positive dying neurons were not increased by IL-17A deficiency in sham animals (hilus: WT 196 ± 31,240 vs. KO 216 ± 48,850; CA1: WT 256 ± 30,150 vs. KO 339 ± 90,870; CA3: WT 236 ± 82,310 vs. KO 267 ± 79,270; Supplementary Figures 3A,B), demonstrating no impact on IL-17A-mediated neuronal death under physiological conditions. These data suggest that IL-17A deletion after pilocarpine-induced SE prevents excitotoxic hippocampal neuronal death.

We also assessed reactive gliosis using immunohistochemistry to Iba1 and GFAP at 3 d after pilocarpine-induced SE (Supplementary Figure 4A). Iba1-immunoreactive area was comparable between IL-17A WT and KO groups in all three hippocampal areas including DG (WT: 30,390 ± 3.901 vs. KO: 34,280 ± 8,467), CA1 (WT: 40,080 ± 6.317 vs. KO: 45,380 ± 11.420) and CA3 (WT: 39,900 ± 5.539 vs. KO: 44,580 ± 10,230) subfields of the hippocampus (Supplementary Figures 4B,C), suggesting that microglial activation was not altered by IL-17A deletion. When the reactive astrocytosis was evaluated, IL-17A deletion did not have an impact on GFAP-immunoreactive areas in DG (WT: 33,230 ± 4,962 vs. KO: 43,870 ± 5,554) and CA1 (WT: 37,680 ± 5,957 vs. KO: 48,290 ± 5,784) subfield of the hippocampus (Supplementary Figures 4D,E), implying no significant difference in the activation of astrocytes by IL-17A deletion in general. However, in CA3 subfield of the hippocampus, GFAP-positive cells were increased in IL-17A KO mice (WT: 18,910 ± 3,779 vs. KO: 34,240 ± 2,676; Supplementary Figures 4D,E), which requires further investigation to appreciate the comprehensive effects of IL-17A on neuroinflammation after acute seizures.

### Interleukin-17A Deletion Reduces the Generation of Hilar Ectopic Granule Neurons

**FIGURE 4:**
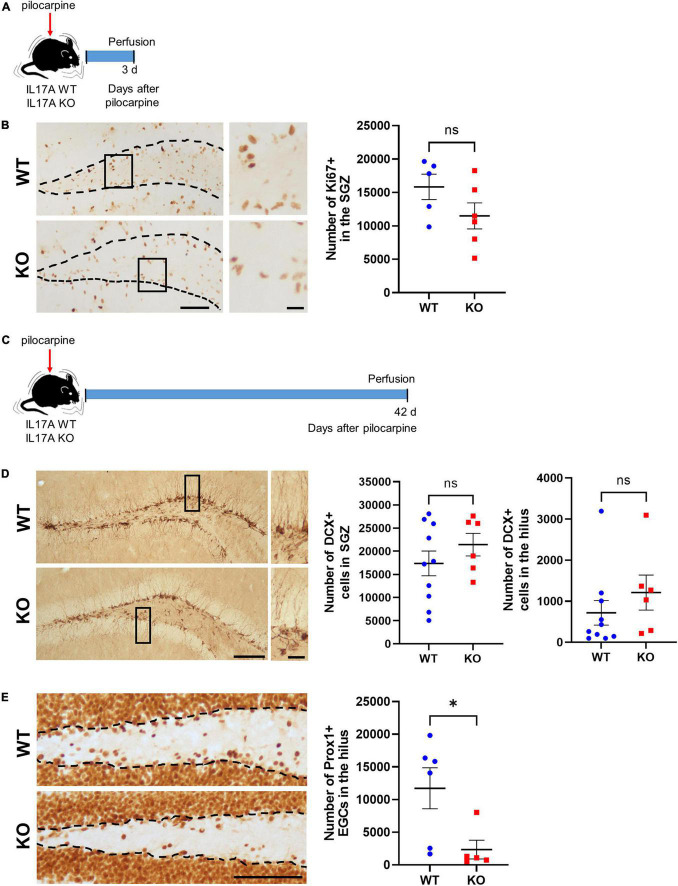
IL-17A deficiency in mice ameliorates seizure-induced aberrant hippocampal neurogenesis. **(A)** Timeline showing the experimental design. **(B)** Representative microscope images and a graph showing immunohistochemistry for Ki67. The rectangles are magnified in the right column. The inner edge of the granule cell layer is indicated as dotted lines. Ki67-immunoreactive cells in the subgranular zone (SGZ) were comparable between IL-17A WT and KO groups. Scale bar = 100 μm for low magnified images, 20 μm for higher magnified images. *n* = 5 (WT) and *n* = 6 (KO). Student’s unpaired *t-*test, *p* = 0.149, *t*(9) = 1.576. **(C)** Timeline showing the experimental design. **(D)** Representative microscope images and graphs showing immunohistochemistry for doublecortin (DCX). The rectangles are magnified in the right column. Scale bar = 100 μm for low magnified images, 20 μm for higher magnified images. *n* = 10 (WT) and *n* = 6 (KO). Detailed statistics are as follows. SGZ: Student’s unpaired *t*-test, *p* = 0.323, *t*(14) = 1.025; hilus: Mann–Whitney *U*-test, *p* = 0.141, *U* = 16,000. **(E)** Representative microscope images and a graph showing immunohistochemistry for prospero homeobox 1 (Prox1). The inner edge of the granule cell layer is indicated as dotted lines. Prox1-immunoreactive cells in the hilus were markedly reduced in IL-17A KO mice. Scale bar = 100 μm. *n* = 6 (WT) and *n* = 5 (KO). Mann–Whitney *U*-test, *p* = 0.017, *U* = 2,000. Data are presented as mean ± SEM. *p < 0.05, NS, not significant.

Since adult neurogenesis is involved in the regulation of anxiety (Revest et al., 2009), we next evaluated whether IL-17A deficiency could modulate seizure-induced hippocampal neurogenesis (Figure 4). Given that hippocampal progenitors are mainly located in SGZ (Parent et al., 1997), we first evaluated Ki67-positive cells in SGZ. Immunohistochemistry of Ki67 showed no significant difference between IL-17A WT and KO groups (WT: 15,826 ± 1,901 vs. KO: 11,484 ± 1,949), demonstrating a similar proliferative activity of neural progenitors at 3 d after pilocarpine-induced SE (Figures 4A,B). We then assessed newly generating neurons in SGZ and seizure-induced mis-migrating cells to the hilus using DCX staining (Walter et al., 2007). The number of DCX-expressing newborn neurons in SGZ (WT: 17,354 ± 2,677 vs. KO: 21,416 ± 2,446) and the hilus (WT: 717,600 ± 300.600 vs. KO: 1,212,000 ± 426,400) was also comparable between the two groups, although there was a slightly increasing trend in IL-17A KO mice (Figures 4C,D). However, IL-17A KO mice exhibited a significant reduction in the number of hilar Prox1-immunoreactive cells (WT: 11,720 ± 3,134 vs. KO: 2,342 ± 1,431; Figure 4E), showing fewer ectopic granule neurons (EGCs) by IL-17A deficiency. When we further analyzed the hippocampal neurogenesis in sham IL-17A WT and KO animals, we found no differences in the basal proliferation of neural progenitors (WT: 4,680 ± 575.400 vs. KO: 4,998 ± 689.900), ongoing hippocampal neurogenesis (SGZ: WT 19,481 ± 1,553,000 vs. KO 16,344 ± 838.400; hilus: WT 85,710 ± 26,630 vs. KO 68,000 ± 19,020), and the hilar EGCs (WT: 760 ± 178,500 vs. KO: 544 ± 97,720) by IL-17A deletion (Supplementary Figures 3C–F). Collectively, our results demonstrate that IL-17A deletion after pilocarpine-induced SE could alleviate seizure-induced generation of hilar EGCs.

## Discussion

The identification of molecular players that can prevent seizure-induced hippocampal pathologies may provide essential information for elucidating the basic mechanisms underlying epilepsy-associated anxiety, as the hippocampus is one of the key regions regulating affective states and anxiety (Revest et al., 2009; Engin et al., 2016; Jimenez et al., 2018). In this study, we demonstrated that IL-17A deletion alleviated elevated anxiety in chronic epilepsy, as evaluated by OFT and EPM test. In line with our findings, previous studies showed that administration of anti-IL-17A or astragaloside IV, an IL-17 inhibitor, ameliorated chronic stress- and stroke-related anxiety, respectively (Sun et al., 2020a; Kim et al., 2021), suggesting a common role of IL-17A in anxiety that occurs in various CNS diseases. Interestingly, we and others reported no impact of IL-17A on anxiety-like behaviors under physiological conditions (Ribeiro et al., 2019; Brigas et al., 2021), although there was a study showing IL-17A-mediated enhanced alertness (Alves de Lima et al., 2020). Taken together, our results identified IL-17A as a novel biomarker for mediating heightened anxiety in chronic epilepsy.

The epileptic IL-17A KO mice in this study showed a reduction in hilar EGCs without affecting the proliferation of neural progenitors or the generation of adult-generated neurons in the hippocampus. IL-17A has shown versatile effects on the different stages of physiological adult neurogenesis. For example, several studies demonstrated that IL-17A impairs the proliferation of progenitors (Cui et al., 2019) and the production of newly generated neurons (Liu et al., 2014), while other studies showed no difference in the proliferation (Rueda et al., 2018; Tfilin and Turgeman, 2019; Willinger and Turgeman, 2022) and conflicting results on neurogenesis (Tfilin and Turgeman, 2019; Willinger and Turgeman, 2022). These controversial findings are also reported in multiple brain diseases including stroke, Down syndrome, and post-traumatic stress disorder (PTSD), i.e., (1) anti-proliferative effects by IL-17A in Down syndrome and PTSD vs. inhibitory or no effects on cell proliferation in stroke, and (2) inhibition of neurogenesis in PTSD vs. inhibition or promotion of neurogenesis in stroke (Lin et al., 2016; Rueda et al., 2018; Sun et al., 2020a,b; Willinger and Turgeman, 2022). These results suggest that the neurobiological response to IL-17A may be differentially regulated depending on different microenvironmental stimuli. Supporting this hypothesis, complex contributions of IL-17A against stroke-induced adult neurogenesis have been proposed to be associated with different temporal IL-17A expression patterns, different IL-17A secreting cell types, different target cells or different regions of the brain (Lin et al., 2016; Sun et al., 2020a,b). Thus, further studies are warranted to elucidate the IL-17A-mediated intricate molecular pathways that can result in different outcomes on injury-induced adult hippocampal neurogenesis.

Surprisingly, there is no conclusive study showing the cause-and-effect relationship between seizure-induced aberrant neurogenesis and comorbid anxiety. Only a few studies altering a transcription factor, fosB, have indicated a possible link of increased ectopic cells to increased anxiety (Ohnishi et al., 2011; Yutsudo et al., 2013). fosB KO mice showed an increased number of hilar ectopic cells after kainic acid (KA)-induced acute seizures (Yutsudo et al., 2013), and manifested increased anxiety (Ohnishi et al., 2011), in agreement with our IL-17A data demonstrating that reduced EGC production in IL-17A KO could attenuate epilepsy-associated anxiety. Collectively, our results support the notion that seizure-induced aberrant hippocampal neurogenesis promotes epilepsy-associated anxiety disorder.

Regarding the neuronal excitation regulated by IL-17A, several contradicting results have been reported (Liu et al., 2014; Xu et al., 2018). In mice in which IL-17A was deleted, intrinsic excitability in the hippocampus was increased (Liu et al., 2014), but in the case of IL-17A receptor or γδ T cell deficiency, KA-induced acute seizure onset was markedly delayed (Xu et al., 2018), suggesting a complex role of IL-17A in hippocampal excitability. Furthermore, after pilocarpine-induced SE, our IL-17A KO mice did not exhibit any difference in the progression of acute seizures, i.e., the onset of the first seizure or SE onset (data not shown). Moreover, the frequency and the duration of SRS in chronic epilepsy did not differ between IL-17A WT and KO mice. Considering the report that IL-17A treatment on the hippocampal slices mimicking the epileptogenic period elicited more spikes in the CA1 pyramidal neurons compared to PBS-treated controls (Xu et al., 2018), it is plausible that during the latent period, marginal reduction of basal excitability in IL-17A KO mice may not be enough to suppress the escalated hippocampal excitability after SE, resulting in chronic recurrent seizures. More rigorous research is necessary to conclude the accurate role of IL-17A in hippocampal neuronal excitability in epilepsy.

Another interesting finding in the present study is the hippocampal neuroprotection after pilocarpine-induced SE by IL-17A deficiency. Unlike the conflicting results on the role of IL-17A in seizure-induced aberrant neurogenesis and neuronal excitation, studies have consistently shown the deleterious influence of IL-17A on neuronal death in many diseases including stroke, traumatic brain injury, and experimental autoimmune encephalitis (EAE) (Shichita et al., 2009; Siffrin et al., 2010; Gelderblom et al., 2012; Li et al., 2017). However, given that hilar EGCs were significantly reduced by IL-17A deletion without altering DCX + newborn neurons in our study, which implies an impairment of EGC survival, increased pyramidal neuronal survival against excitotoxic injury in IL-17A KO mice may seem to be a conflicting finding. We speculate that the reason behind this discrepancy may be associated with the cellular developmental status and its different response to intracellular calcium, which is a key signaling ion involved in diverse cellular processes ranging from neurite outgrowth, synaptogenesis, synaptic transmission, cell survival, and excitotoxic cell death (Mattson, 2007). Interestingly, IL-17A-producing Th17 cells increase neuronal intracellular calcium level in a sustained manner, promoting neuronal damage in EAE (Siffrin et al., 2010). It is plausible that the pyramidal neurons in our study may escape from the excitotoxic cell death by decreased calcium influx attributed from the deletion of IL-17A. However, for the newly generated EGCs that are still developing, calcium signal is crucial for synaptic integration and granule neuronal survival (Ge et al., 2008; Kempermann et al., 2015). Thus, IL-17A deletion may provide insufficient calcium signal to developing granule neurons, thereby reducing the survival of EGCs. However, more rigorous research will be required to corroborate this hypothesis in future studies.

In summary, we showed that IL-17A deletion could alleviate epilepsy-associated anxiety disorder, possibly by reducing hilar EGC production and excitotoxic neuronal death. Our findings can provide the basis for understanding epilepsy-associated psychiatric comorbidities, especially anxiety disorder.

## Data Availability Statement

The original contributions presented in the study are included in the article/Supplementary Material, further inquiries can be directed to the corresponding author/s.

## Ethics Statement

The animal study was reviewed and approved by the Institutional Animal Care and Use Committee at the Catholic University of Korea (Approval number: CUMS-2020-0342-01, 2020-0103-04).

## Author Contributions

K-OC conceived and designed the study. M-LC provided study materials and helped to revise the manuscript. I-YC and K-OC performed the experiments, analyzed the data, and wrote and revise the manuscript. All authors contributed to the article and approved the submitted version.

## Conflict of Interest

The authors declare that the research was conducted in the absence of any commercial or financial relationships that could be construed as a potential conflict of interest.

## Publisher’s Note

All claims expressed in this article are solely those of the authors and do not necessarily represent those of their affiliated organizations, or those of the publisher, the editors and the reviewers. Any product that may be evaluated in this article, or claim that may be made by its manufacturer, is not guaranteed or endorsed by the publisher.
