# Supplementary figures and images for: Interleukin-17A Mediates Hippocampal Damage and Aberrant Neurogenesis Contributing to Epilepsy-Associated Anxiety
Source: Front Mol Neurosci. 2022 Jul 6;15:917598. doi: 10.3389/fnmol.2022.917598 (PMC9298510; doi:10.3389/fnmol.2022.917598)

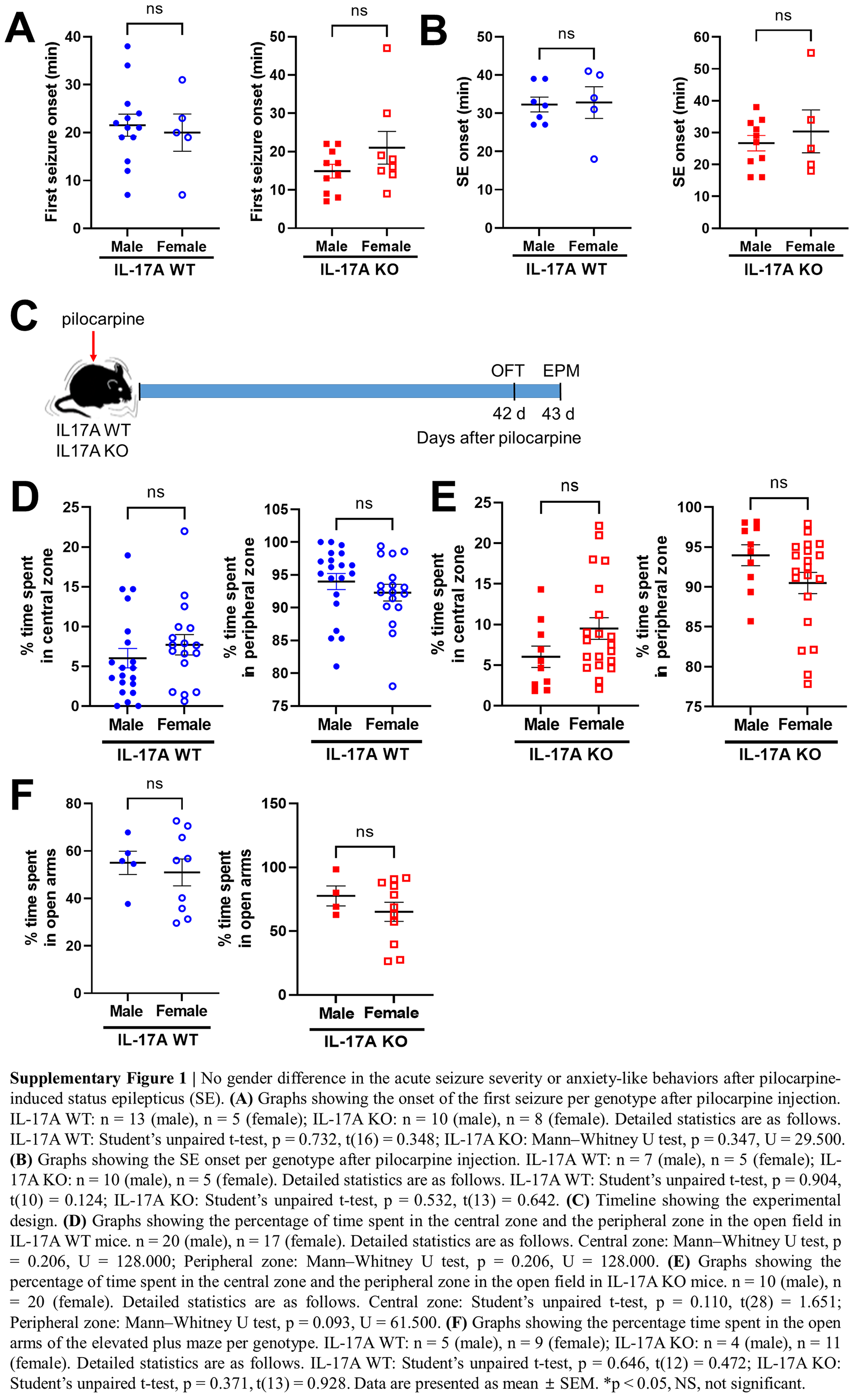

Supplement: Supplementary file 1 [file Image_1.TIF]

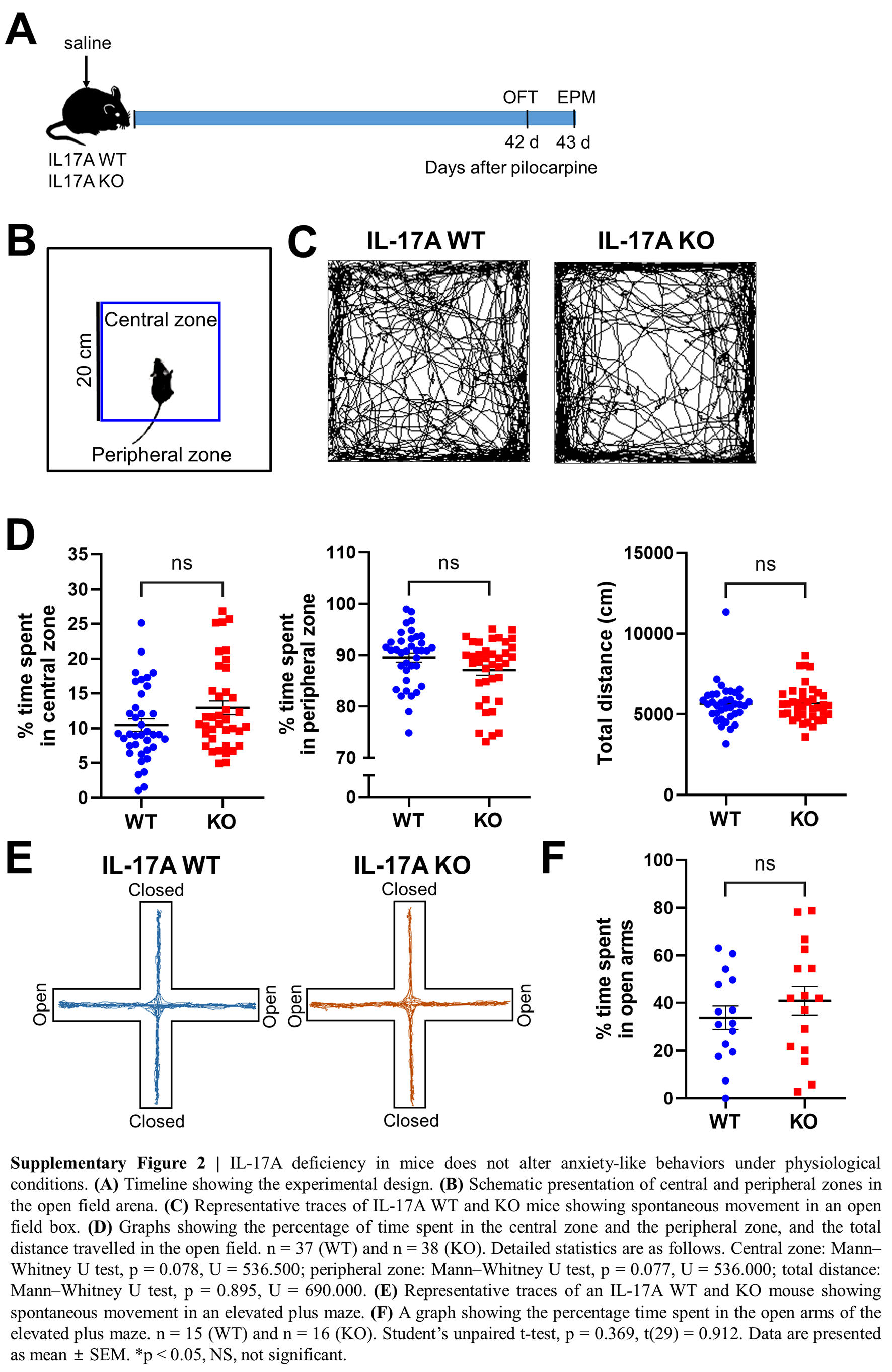

Supplement: Supplementary file 2 [file Image_2.TIF]

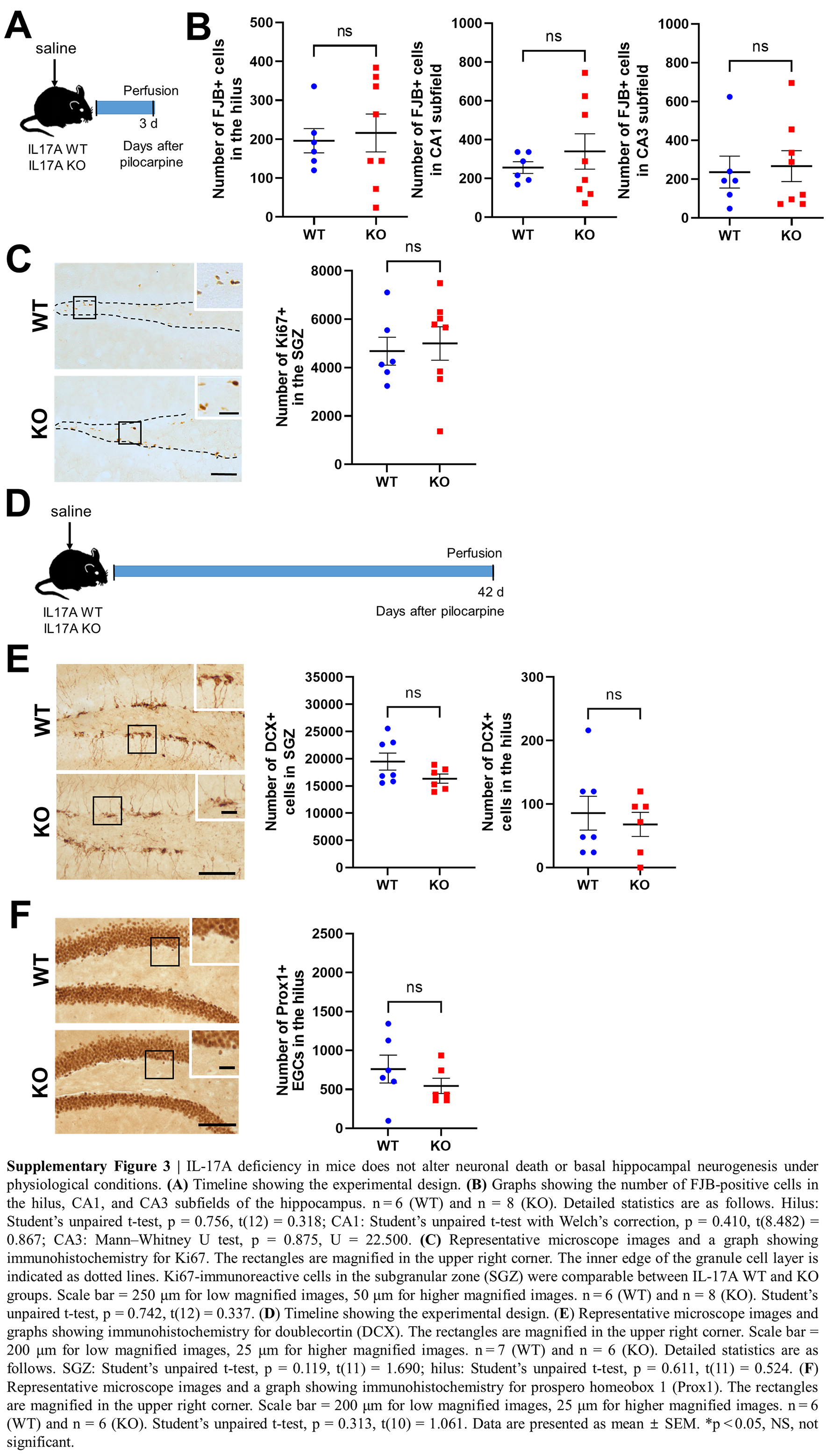

Supplement: Supplementary file 3 [file Image_3.tif]

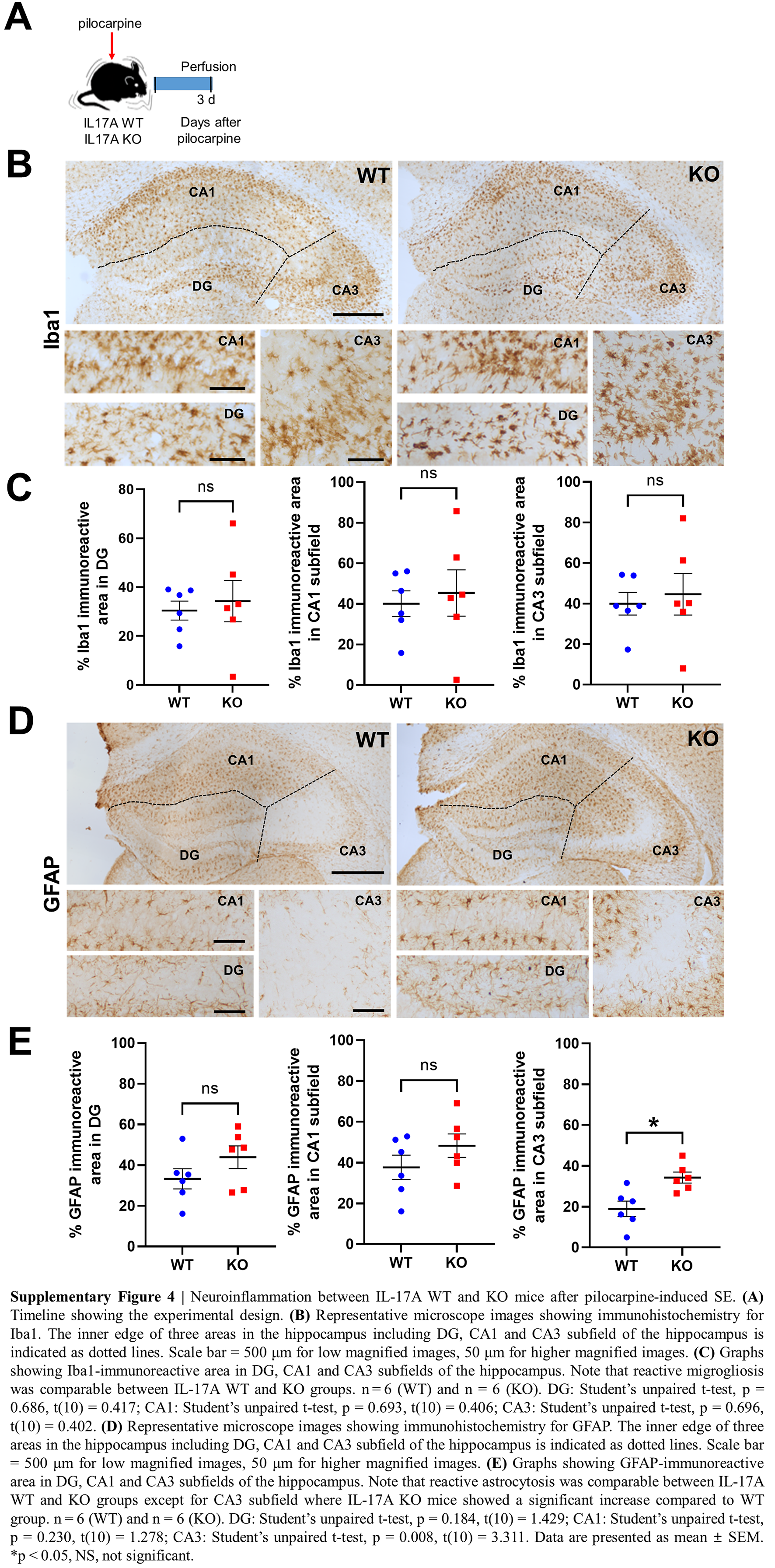

Supplement: Supplementary file 4 [file Image_4.TIF]
